# Testing the effect of pollen exine rupture on metabarcoding with Illumina sequencing

**DOI:** 10.1371/journal.pone.0245611

**Published:** 2021-02-02

**Authors:** Stephanie J. Swenson, Birgit Gemeinholzer

**Affiliations:** Systematic Botany, Justus Liebig University Gießen, Giessen, Germany; Austrian Federal Research Centre for Forests BFW, AUSTRIA

## Abstract

Pollen metabarcoding has received much attention recently for its potential to increase taxonomic resolution of the identifications of pollen grains necessary for various public health, ecological and environmental inquiry. However, methodologies implemented are widely varied across studies confounding comparisons and casting uncertainty on the reliability of results. In this study, we investigated part of the methodology, the effects of level of exine rupture and lysis incubation time, on the performance of DNA extraction and Illumina sequencing. We examined 15 species of plants from 12 families with pollen that varies in size, shape, and aperture number to evaluate effort necessary for exine rupture. Then created mock communities of 14 of the species from DNA extractions at 4 levels of exine rupture (0, 33, 67, and 100%) and two levels of increased lysis incubation time without exine rupture (2 or 24 hours). Quantities of these DNA extractions displayed a positive correlation between increased rupture and DNA yield, however increasing time of lysis incubation was associated with decreased DNA yield. Illumina sequencing was performed with these artificial community treatments with three common plant DNA barcode regions (*rbcL*, ITS1, ITS2) with two different primer pairings for ITS2 and *rbcL*. We found decreased performance in treatments with 0% or 100% exine rupture compared to 33% and 67% rupture, based on deviation from expected proportions and species retrieval, and increased lysis incubation was found to be detrimental to results.

## Introduction

Accurate identification of the plant species present in mixed environmental pollen samples has important applications across several diverse disciplines including pollination ecology, forensics, paleobotany, and airborne allergen monitoring. Traditional pollen identification based on external morphology of the pollen grain is time consuming and requires a high level of training, and for many taxa, identification below the family level is not possible. Due to these limitations, metabarcoding has become an exciting alternative technique with the potential to provide faster identification of pollen, often to the species level. Despite this potential, metabarcoding in general and pollen metabarcoding in particular are not without complications. These complications must be accounted for, or overcome, before this technique can be reliably applied to large-scale biomonitoring or other programs that could affect conservation management, public health or government policy.

All metabarcoding programs consist of collection of environmental DNA samples, DNA extraction, amplification and sequencing. The methods chosen for each of these steps are most often dictated by user preference, cost, equipment available, and current level and/or availability of technology rather than the most rigorously tested and optimal protocol. Each one of these steps has the potential to skew results or introduce biases that affect downstream processes [1], it is therefore important for the development of metabarcoding programs, to perform comparisons of several methods in order to account for inconsistencies and provide quality controls of future investigations. The most critical aspect of improving metabarcoding performance is therefore the optimization of the initial steps. Each method of collection of environmental pollen samples inherently results in unintended capture of non-pollen plant material or non-plant taxa (e.g. fungal spores or insect fragments) and/or uses liquids, chemicals, or adhesives that can inhibit DNA extraction and amplification. There are very few ways in which pollen collection methods can be improved upon; therefore, optimizing the extraction method to overcome these potential inhibitors is the logical starting point for improvement and standardization of pollen metabarcoding.

The initial step of extraction of pollen DNA from non-germinated pollen is to break the strong outer wall of the pollen grain, called the exine, either by chemical or mechanical methods. This exine is composed of a biopolymer known as sporopollenin that is highly resistant to chemical, physical, and enzymatic degradation [2]. Breakage of the exine with the underlying cell walls is believed to be necessary to free the genetic material from these protective outer layers [3].

Biological samples that are heavily encased and not effectively disrupted by chemical lysis alone can undergo mechanical homogenization prior to DNA extraction in order to increase yield [4]. This is commonly performed by bead milling, which has maximum efficiency when the size and composition of the material being ground is matched to the container and grinding material implemented [4]. Undoubtedly, efficiency of exine rupture will be in part based on size of the individual pollen grains and the size and quantity of beads used; however, the strength and elasticity of the pollen grains’ exine is highly variable. This variable strength is due to many factors beyond the sporopollenin composition, such as, the distribution of structural materials into smaller subunits, extra deposition of sporopollenin at points of greater stress, creation of striated patterns that distribute force, and the geometric arrangement of the apertures [5]. Pollen apertures are areas of reduced exine deposition, the patterns of which are species specific and can vary in size, shape, number and margination. They are the usual site of pollen tube development and likely have an important role in interaction between a pollen grain and the environment [2].

While disruption of the pollen exine, gametophytic cell walls, and nuclear membranes are necessary for efficient DNA extraction, the method implemented must also avoid degrading the DNA in the process. Simel et al. [3] evaluated several chemical and mechanical methods to achieve this and determined bead milling, to be the most efficient method to rupture the exine while maintaining abundant non-degraded DNA. This study examined each species individually however, and there is little information available concerning how to best perform this on the mixed species samples examined in metabarcoding. Due to the differential resiliency of the species’ exines in mixed species samples, any method of exine maceration could result in some species’ genetic material being retained in the pollen grain while other species’ DNA would be over processed and sheared. This differential processing has the potential to greatly skew the diversity of species returned from sequencing.

Likely due to the results of Simel et al. [3], the majority of pollen metabarcoding studies have utilized microbead maceration as the method of exine rupture. However, there is little consistency among studies in the size, material or quantity of beads utilized, or in the time and frequency of agitation employed (Table 1). In addition, there has been little examination into the effect this step has on quality and quantity of DNA produced, and the information that does exist is contradictory. Pornon et al. [6] saw no effect of bead milling on quantity or quality of DNA, while Prosser and Hebert [7] found unground samples or those ground with 3 mm tungsten beads produced only 1% of the DNA concentration of those ground manually with 100 mg of 0.3 mm sterile sand, and Leontidou et al. [8] showed a significant increase in yield (p < 0.001) with steel 5 mm beads over glass 0.5 mm beads. To date, no study has used mock communities to examine the effect of exine rupture methodology on species retrieval from sequencing.

**Table 1:** Methods of exine homogenization implemented in previous pollen studies.

Method of homogenization	Bead size and duration of processing	Reference
Homogenization method not mentioned		[9–15]
Homogenization method other than bead milling		[16, 17]
Bead milling performed as homogenization method	Bead size and duration of processing not specified	[18–22]
	Beads 3–3.2 mm beads, 3–4 minutes processing in a bead mill	[23–28]
	Beads ≤ 1.0 mm, two minutes processing in a bead mill	[29–32]
	Beads ≤ 1.0 mm, 5–10 mins of manual processing	[33, 34]
	Beads a mix of sizes, two minutes processing in a bead mill	[8, 35, 36]
Bead milling performed as homogenization method, and bead size and/or duration tested		[6–8]

In addition to mechanical disruption of the pollen exine, duration of incubation in lysis buffer in the DNA extraction protocol could also influence final DNA yield. It is possible this step in DNA extraction could greatly influence DNA yield from thin-walled pollen grain or those with multiple apertures that are potentially more susceptible to lysis buffer than thick walled or uniporate species.

In this study, we examined the effects of level of exine rupture and lysis incubation time on the performance of DNA extraction and Illumina sequencing. We examined 15 species from 12 plant families with pollen that varies in size, shape, and aperture number and employed different bead mill bead sizes and durations of milling to find optimal rupture conditions for each species. DNA of 14 of the species from this rupture experiment were then extracted at four levels of exine rupture (0, 33, 67, and 100%) and two levels of longer duration of lysis incubation without exine rupture (2 or 24 hours). We measured quantity of these DNA extractions to test whether a positive correlation between exine rupture levels and quantity of DNA exists for these species. From these extractions, we performed Illumina MiSeq sequencing with three common plant DNA barcode regions (*rbcL*, ITS1, ITS2) as well as two different primer pairings for ITS2 and *rbcL*, in order to examine 1) the relationship between increased levels of exine rupture and lysis time on proportions of reads produced and 2) to evaluate the performance of common plant barcodes and primer pairings with a mock community containing equal quantities of pollen grains as starting material.

## Materials methods

### Exine rupture quantification

Fifteen species of pollen representing 12 families varying in size, shape, and aperture number were purchased (Bonapol a. s. Czech Republic) or collected by hand (Table 2). Species included represent the highest taxonomic and morphological breadth that could be obtained in high quantities without contamination from other species or non-pollen plant material. Four replicates of each species were made by adding approximately 1 mg of pollen to 1 mL 50% glycerol solution [37] in a 2 mL SafeSeal microcentrifuge tube (Sarstedt AG & Co. KG).

**Table 2:** Morphology and collection information for species used in this study and optimal bead mill conditions for near complete exine rupture.

Species	Family	Species code	Shape	Size Category	Number of Apertures	Collection source	Optimal bead size (mm)	Time to 95% rupture (seconds)
*Abies concolor*	Pinaceae	AC	Saccate	Large (51–100 μm)	1	Bonapol	1.4	112
*Alnus glutinosa*	Betulaceae	AG	Oblate	Medium (26–50 μm)	5	Bonapol	2.8	605
*Brassica napus*	Brassicaceae	BN	Prolate	Medium (26–50 μm)	3	Bonapol	1.4	249
*Chenopodium album*	Amaranthaceae	CA	Spheroidal	Medium (26–50 μm)	25–70+	Bonapol	2.8	493
*Cupressus sempervirens*	Cupressaceae	CS	Spheroidal	Medium (26–50 μm)	0	Bonapol	1.4	231
*Dactylis glomerata*	Poaceae	DG	Spheroidal	Medium (26–50 μm)	1	Hand collected, Giessen, Germany	1.4	325
*Picea sp*.	Pinaceae	Pic	Saccate	Large (51–100 μm)	1	Hand collected, Giessen, Germany	2.8	211
*Plantago lanceolata*	Plantaginaceae	PL	Spheroidal	Small (10–25 μm)	9–15	Hand collected, Aschaffenburg, Germany	2.8	502
*Populus* sp.	Salicaceae	Pop	Spheroidal	Medium (26–50 μm)	0	Hand collected, Aschaffenburg, Germany	1.4	66
*Rumex acetosella*	Polygonaceae	RA	Spheroidal	Small (10–25 μm)	3	Bonapol	1.4	123
*Salix caprea*	Salicaceae	SC	Spheroidal	Small (10–25 μm)	3	Bonapol	1.4	482
*Taxus baccata*	Taxaceae	TB	Elliptic	Small (10–25 μm)	0	Hand collected, Rhodes, Greece	1.4	69
*Tilia platyphyllos*	Tiliaceae	TP	Oblate	Small (10–25 μm)	3	Bonapol	5.0	323
*Ulmus glabra*	Ulmaceae	UG	Spheroidal	Medium (26–50 μm)	5–6	Bonapol	2.8	660
*Zea mays*	Poaceae	ZM	Spheroidal	Large (51–100 μm)	1	Bonapol	5.0	114

Four bead size treatments were created by addition of 1) 1 x 5 mm steel bead, 2) 3 x 2.8 mm ceramic beads, 3) 1 g 1.4 mm ceramic beads, or 4) 0.5 g 0.5 mm glass beads to the 1 mg/mL sample. These sizes and quantities were chosen as they generally correspond to sizes used in previous publications (Table 1).

Intact pollen grains were counted in 20 microscope fields by transferring 24 μL of the pollen solution (2 x 12 μL) to a hemocytometer after 6 durations in a Retsch MM400 bead mill (0, 30, 60, 120, 300, and 600 seconds) or until no intact pollen grains could be found. From these time interval counts, time to 33%, 67%, 95%, and 100% rupture were calculated and best bead size was determined to be the size that resulted in the shortest duration to 95% rupture.

### DNA extraction

Fourteen of the above species (*Cupressus sempervirens* L. was eliminated due to presence of non-pollen plant material in sample) were suspended as single species in sterile 50% glycerol and 10 replicate counts of 10 μL were performed in order to create 18 replicate aliquots of 10,000 pollen grains for each species. From these 18 replicates, 6 treatments with 3 replicates (Table 3) were created by implementing the optimal bead mill conditions determined above. DNA extraction was performed with Macherey Nagel Nucleospin food kit following the instructions for honey with 400 μL lysis buffer and a final elution volume of 50 μL. Mixed species DNA extractions were created by combining equal volumes of each single species extractions for a final creation of 3 replicates of each of the 6 exine rupture treatments. The DNA quantity of these mixed species extractions were quantified with Qubit™ 4 fluorometer using the dsDNA HS Assay kit.

**Table 3:** Experimental treatment conditions.

Estimated level of exine rupture	Duration of lysis incubation	Code for each replicate
0%	1 hour	0.1, 0.2, 0.3
0%	2 hours	2h.1, 2h.2, 2h.3
0%	24 hours	24h.1, 24h.2, 24h.3
33%	1 hour	33.1, 33.2, 33.2
67%	1 hour	67.1, 67.2, 67.3
100%	1 hour	100.1, 100.2, 100.3

### Barcode selection

In order to separate the effect of exine rupture from PCR bias, three common plant barcodes (ITS1, ITS2, and *rbcL*) were chosen. ITS2 and *rbcL* were performed with two different primer pairings to further examine PCR bias and performance with a mock community (Table 4). The primer pairs for *rbcL* create regions that accommodate the read length limitations of Illumina sequencing but they differ in the area of coverage. The ITS2 primer pairs differ in that the combination of ITS2-2SF/ITS4 (referred to hereafter as ITS2 universal primers) is a universal pairing that will also amplify fungus, the combination of ITS-3p62plF1/ITS-4unR1 (referred to hereafter as ITS2 plant specific primers) were created to target plant taxa more specifically in order to minimize the occurrence of false negatives.

**Table 4:** Primers used in this study.

*Barcode*	Primer name	Primer sequence	Reference
*rbcL*	rbcL2	TGGCAGCATTYCGAGTAACTC	[38]
rbcL	rcbLaR	CTTCTGCTACAAATAAGAATCGATCTC	[39]
rbcL	rbcLaF	ATGTCACCACAAACAGAGACTAAAGC	[40]
rbcL	rbclr506	AGGGGACGACCATACTTGTTCA	[41]
ITS1	ITS-1unF1	GGAAGKARAAGTCGTAACAAGG	Andreas Kolter (pers. comm.)
ITS1	ITS-4unR1	GCCDAGATATCCRTTGYCRRGAG	Andreas Kolter (pers. comm.)
ITS2	ITS2-2SF	ATGCGATACTTGGTGTGAAT	[42]
ITS2	ITS4	TCCTCCGCTTATTGATATGC	[43]
ITS2	ITS-3p62plF1	ACBTRGTGTGAATTGCAGRATC	Andreas Kolter (pers. comm.)
ITS2	ITS-4unR1	TCCTCCGCTTATTKATATGC	Andreas Kolter (pers. comm.)

### PCR and sequencing

PCR was performed in three replicates per sample with an adaptation of the Canadian Centre for DNA Barcoding Platinum® Taq PCR protocol [44], with the addition of 0.25 μL of BSA and 1.25 μL of 50% DMSO in a total volume of 12.5 μL per reaction. Primers utilized are listed in Table 3. PCR cycling conditions were: 95°C for 3 minutes; followed by 35 cycles of 95°C for 30 seconds, 50°C for 30 seconds, 72°C for 45 seconds; and a final extension of 72°C for 10 minutes. A PCR negative control was included for each PCR. Following PCR cycling the three replicates were combined and purified with Thermo Scientific™ Exonuclease I. The pooled replicates of non-indexed PCR products were sent to LGC Genomics GmbH where indices were added and sequencing was performed with Illumina protocol in the 2 × 300 bp format.

Raw reads of ITS2 were filtered and paired with the pipeline of Sickle et al. [20] and *rbcL* reads were filtered and paired with the pipeline of Bell et al. [21], both of these pipelines perform pairing with QIIME v.1.8.o [45] with default parameters, and low quality reads (<Q20, <150 bp, ambiguous base pairs) are removed with USEARCH v8 [46]. Both *rbcL* and ITS2 were classified with the UTAX algorithm and the database created for the respective pipelines. ITS1 reads were filtered and paired with the same parameters of ITS2 and *rbcL*, however OTU clustering was performed with USEARCH v11 [46] and OTUs were identified with the SINTAX algorithm [47] and the curated reference database PlaniTS1 [48].

### Data analysis

A two-way ANOVA was performed using R software [49] (R Core Team 2016) to test for association between pollen grain size and number of apertures and time to 95% rupture.

Barcode and/or primer pair performance were evaluated by the number of species from the mock community returned from sequencing runs. Correct genus assignment was accepted as indication of species presence. A species was considered present when five or more reads per species were recovered in at least two of the three replicates in one or more exine rupture treatment.

In evaluation of the effect of exine rupture treatment on read proportions returned, any presence (i.e. singletons reads and above) of the known species was counted. The resulting read proportion of a species was subtracted from the expected read proportion and then totaled for each treatment and replicate. The optimal exine rupture level for each barcode was determined to be the treatment that produced the lowest additive value of deviation from expected proportion.

## Results

### Exine rupture

**Fig 1:**
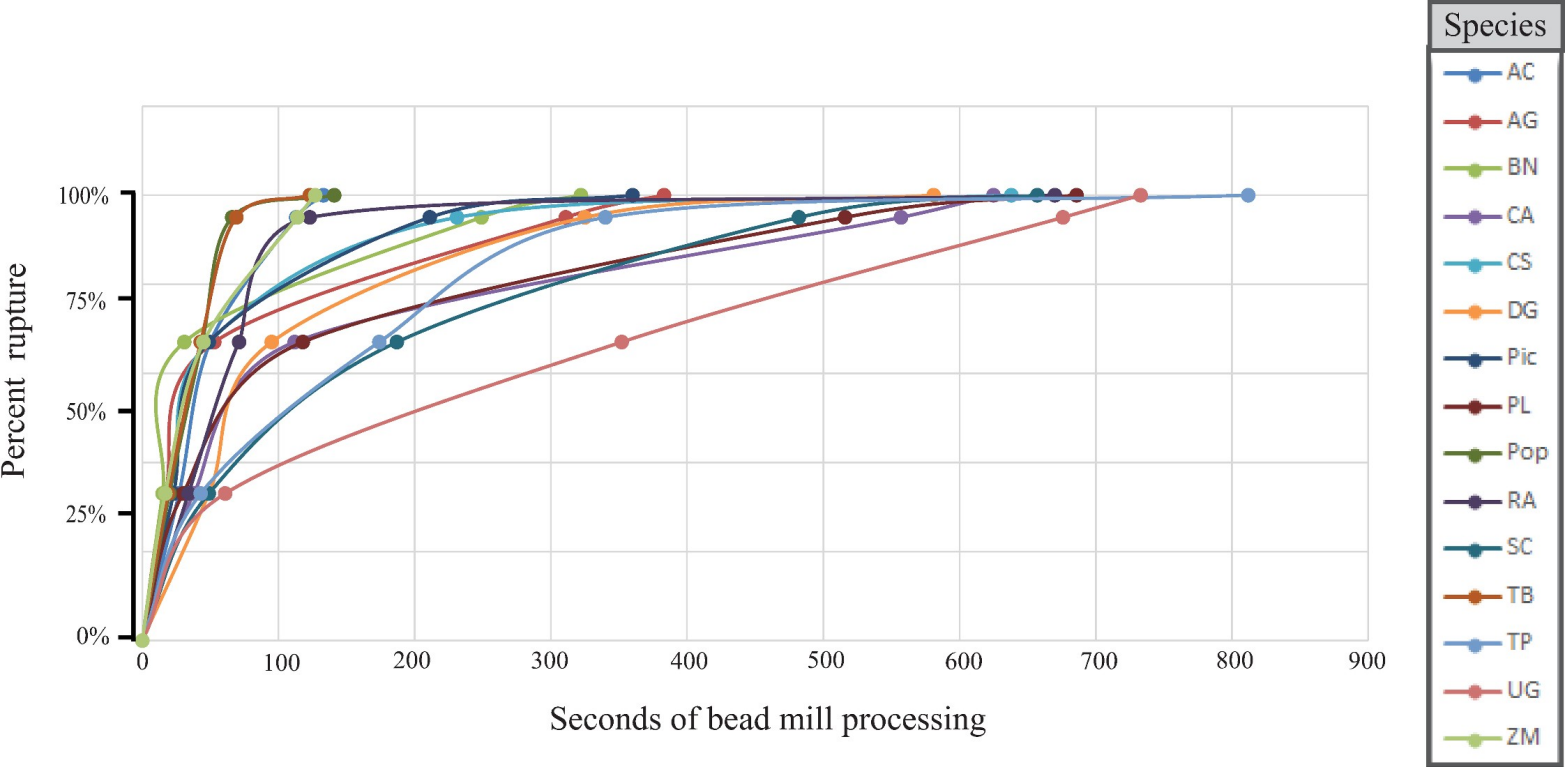
Curves produced from projected time to complete rupture for all species when optimal bead size is utilized. Species codes are listed in Table 2.

Time to 95% rupture varied widely among species (mean = 304.3 seconds, Standard deviation = 199.9, Range = 594) (Fig 1). No association was recovered between pollen grain size and time to 95% rupture, however the association of time to 95% rupture and aperture number was significant (ANOVA; p < 0.001).

For the majority of species rupture with 1.4 mm beads resulted in the fastest time to 95% (8 of 15) while rupture with 2.8 mm beads resulted in the fastest time to 95% for five species (Table 2). Rupture with a single 5.0 mm bead resulted in the fastest time to 95% for two species (*Tilia platyphyllos* Scop. and *Zea mays* L.) however in both of these species 1.4 mm and 2.8 mm beads resulted in nearly equivalent times. Rupture with 0.5 mm beads did not result in most efficient rupture in any of the species examined and in two instances projected time to 95% rupture exceeded 10 minutes.

### DNA yield

Quantity scores, as the average of the 3 replicates, increased threefold when rupture level was increased from 0% to 100% (0%(1 hour lysis) = 0.531 ng/μl, 0%(2 hour lysis) = 0.588 ng/μl, 33 = 0.808 ng/μl, 67 = 1.335 ng/μl, 100 = 1.512 ng/μl), however the 24 hour lysis with 0% exine rupture recovered very low quantities (0.222 ng/μl) and was not successful in amplification.

### Metabarcoding results, barcode performance

ITS2 with either primer pairing returns all species (except *Zea mays*) above the threshold (Table 5). One primer pairing of *rbcL* (rbcLaF, rbclr506) excludes only *Taxus baccata* L. and *Zea mays*, while the other pairing (rbcL2, rbclaR) returns only five of the 14 species present in this mock community. *Tilia platyphyllos* and *Zea mays* were not recovered above the threshold for ITS1 and due to the long length of ITS1 in most gymnosperms, *Abies concolor* (Gord. et Glend.) Lindl.) ex Hildebr., *Picea sp*., *and Taxus baccata* were not recovered from paired reads but could be evaluated if only single read directions are considered.

**Table 5:** Species retrieved from Illumina MiSeq run above the threshold of a minimum of five reads in at least two replicates of at least one exine rupture treatment.

Species	Barcode and Primer Pairs				
	ITS1	ITS2 (ITS2-2SF/ITS4)	ITS2	*rbcL* (rbcL2/rbcLaR)	*rbcL* (rbcLaF/rbcLr506)
*Abies concolor*		✓	✓	✓	✓
*Alnus glutinosa*	✓	✓	✓		✓
*Brassica napus*	✓	✓	✓		✓
*Chenopodium album*	✓	✓	✓		✓
*Dactylis glomerata*	✓	✓	✓		✓
*Picea* sp.		✓	✓	✓	✓
*Plantago lanceolata*	✓	✓	✓		✓
*Populus* sp.	✓	✓	✓	✓	✓
*Rumex acetosella*	✓	✓	✓		✓
*Salix caprea*	✓	✓	✓	✓	✓
*Taxus baccata*		✓	✓	✓	
*Tilia platyphyllos*		✓	✓		✓
*Ulmus glabra*	✓	✓	✓		✓
*Zea mays*					

### Metabarcoding results, evaluation of read proportions

All barcodes and primer pairings deviated from the expectation of equal proportion of each species at expected value of 0.0714. Due to the low number of species returned with the rbcL2/rbcLaR primer pairs, the data were eliminated from this evaluation. The treatments with 0% rupture plus increased duration of lysis incubation (2 and 24 hours) were also eliminated from further analyses due to suboptimal sequencing results or amplification failure that confounded comparable results.

**Fig 2:**
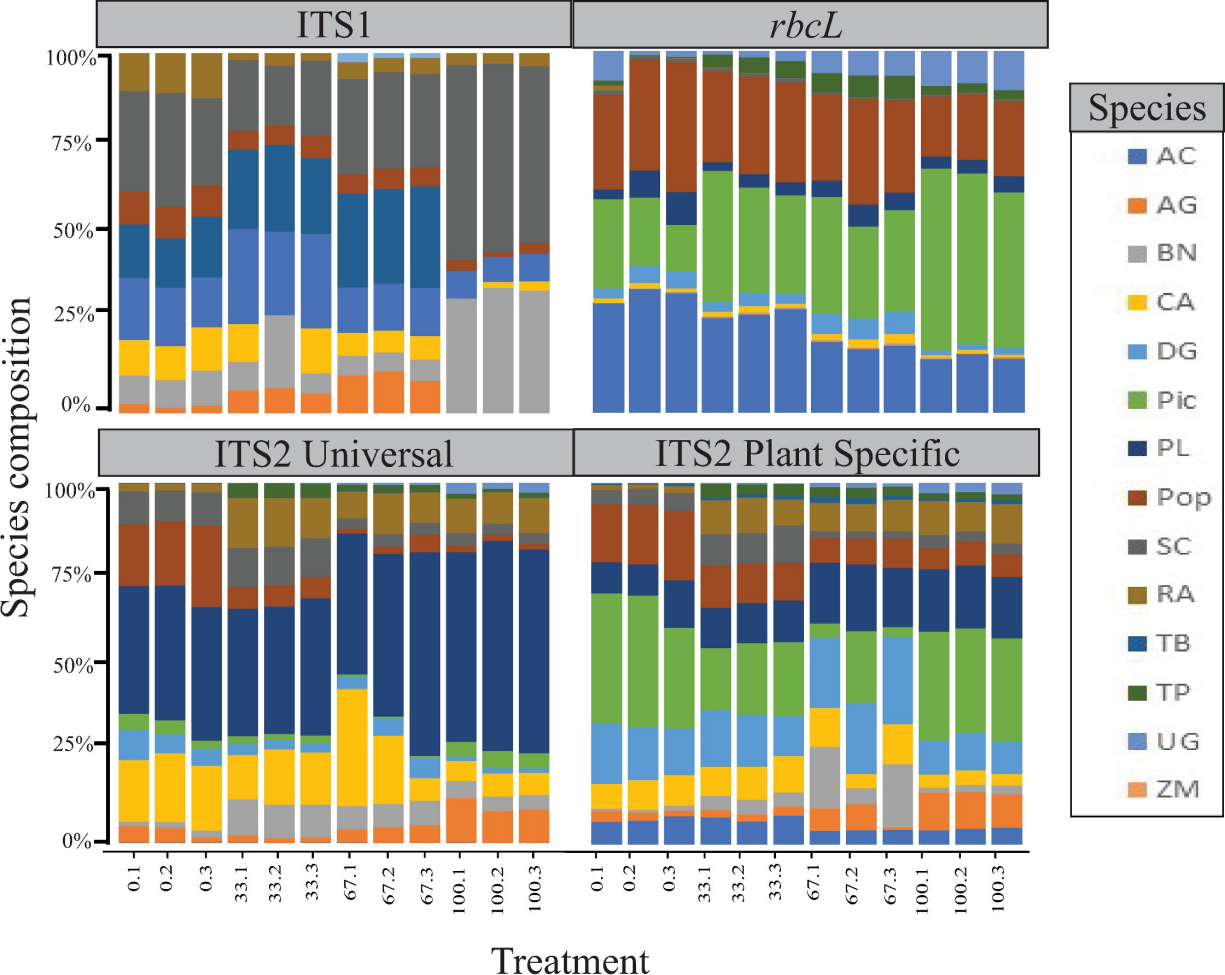
Species composition for all replicates of each treatment, codes for treatments are listed in Table 3 and species codes are listed in Table 2.

No general trend that persisted across all primer pairs, treatments and species was recovered (Table 6, Fig 2), however there is little variation between treatment replicates. *Zea mays* is uniformly nearly eliminated across all primer pairs and is never present in more than two reads, *Taxus baccata* is also represented in very low read counts, often not above the five read threshold. No species is present at or near expected proportions across all treatments in all barcodes or primer pairs.

**Table 6:** Trends of individual species’ proportion of reads returned from Illumina sequencing of the different level of exine rupture treatments.

Trend	marker	species
Species is present above expected proportion across all treatments	*rbcL*(rbcLaF/rbcLr506)	*Picea sp*.
		*Populus sp*.
	ITS1	*Rumex acetosella*
	ITS2 (Universal)	*Plantago lanceolata*
	ITS2 (Plant specific)	-
Species is present below expected proportion across all treatments	*rbcL* (rbcLaF/rbcLr506)	*Taxus baccata*
		*Zea mays*
	ITS1	*Abies concolor*
		*Picea sp*.
		*Taxus baccata*
		*Tilia platyphyllos*
		*Ulmus glabra*
		*Zea mays*
	ITS2 (Universal)	*Abies concolor*
		*Taxus baccata*
		*Zea mays*
	ITS2 (Universal)	*Taxus baccata*
		*Zea mays*
Species is present at or near expected proportion across all treatments	*rbcL* (rbcLaF/rbcLr506)	*Plantago lanceolata*
	ITS1	-
	ITS2 (Universal)	-
	ITS2 (Plant specific)	-
Present with high deviation from expected proportion (either high or low) and approaches expected proportion at higher levels of rupture.	*rbcL* (rbcLaF/rbcLr506)	*Brassica napus Chenopodium album Dactylis glomerata Rumex acetosella*
		*Salix caprea*
		*Tilia platyphyllos*
	ITS1	*Alnus glutinosa*
		*Chenopodium album*
		*Dactylis glomerata*
	ITS2 (Universal)	*Alnus glutinosa*
		*Brassica napus*
		*Picea sp*.
		*Ulmus glabra*
	ITS2 (Plant specific)	*Alnus glutinosa*
		*Ulmus glabra*

No species displays the trend of high deviation from expected values (either very low or very high) at 0% rupture and approaching the expected proportion at a higher level of rupture, across all barcodes or primer pairs. There are several instances of this within a single barcode or primer pair (Table 6).

### Proportions evaluation

The mean of values for deviation from expected proportions was lowest in the 0% rupture treatment for ITS1, the 33% rupture treatment for ITS2 (universal and plant specific primers), and the 67% rupture for *rbcL*. Values for standard deviation of replicates indicate low variation in read proportions within individual treatments (Table 7, Fig 2).

**Table 7:** Cumulative deviation from expected proportions totaled for all species in each treatment and replicate.

Barcode	Treatment	Replicate 1	Replicate 2	Replicate 3	Mean	St. Dev.
	0%	1.2094	1.2536	1.2436	1.2356	0.0232
*rbcL*	33%	1.3201	1.2346	1.2376	1.2641	0.0485
(rbcLaF/rbcLr506)	67%	1.0824	1.0136	1.0129	1.0363	0.0399
	100%	1.2538	1.2362	1.2105	1.2335	0.0218
	0%	1.0274	1.0899	1.1689	1.0954	0.0709
ITS2	33%	0.9335	0.9686	0.9604	0.9542	0.0184
(Universal)	67%	1.1533	1.0824	1.0132	1.0830	0.0700
	100%	1.0628	1.0914	1.0806	1.0783	0.0145
	0%	0.9779	0.9738	0.9291	0.9602	0.0271
ITS2	33%	0.6455	0.6748	0.6386	0.6530	0.0192
(Plant specific)	67%	0.7216	0.7411	0.8588	0.7738	0.0742
	100%	0.8197	0.7846	0.7803	0.7949	0.0216
	0%	0.9477	0.9676	0.9564	0.9572	0.0100
ITS1	33%	1.0137	1.1108	1.0407	1.0547	0.0496
	67%	0.9418	0.9837	0.9612	0.9622	0.0210
	100%	1.444	1.4611	1.3872	1.4309	0.0387

## Discussion

### Exine rupture

Our results reflect the extreme variation and species’ specific nature of the strength of the pollen grain exine. No single bead size was most efficient for exine rupture of all of the species examined and no relationship between size of pollen grain and bead size was recovered. This confirms that exine strength is comprised of more factors than surface to volume ratio alone. The two bead sizes that gave the shortest duration to 95% rupture (1.4 mm and 2.8 mm) reflect sizes used in several previous publications (Table 1). We observed a very wide range of time to achieve 95% rupture, from about one minute with *Populus* sp. and *Taxus baccata* to over 10 minutes in *Ulmus glabra* and *Alnus glutinosa*. This further illustrates the extreme variation in exine durability and architecture that occurs as a result of environmental pressures and strategies of dispersal, reproduction, and anti-desiccation [2]. We found an association between increased aperture number and increased time to 95% rupture corroborating the ideas of Payne [5] that arrangements and increased quantities of apertures contribute to the resistance of the exine to breakage.

### Effect of exine rupture on DNA extraction quality

Much emphasis has been placed on the importance of the bead mill process in pollen DNA extraction with very little supporting evidence for the necessity of this process. While we did find that increased rupture did increase DNA quantity from 0.531 ng/μl to 1.512 ng/μl in our mixed samples, we expected the effect to be much more pronounced than observed. A complication in our evaluations is the low quantity of DNA that was produced from the extractions of 10,000 pollen grains, higher quantities of starting material might have the potential to better illuminate the changes in quantity. An additional complication is that quantity was measured after species mixes were created and we are unable to evaluate the effect of increasing rupture on each species as an individual. It is possible that the positive effect of exine rupture on quantity scores will be reduced in multiporate species that are potentially more permeable to lysis buffer or thin walled species that are prone to breakage with liquid contact alone. We expect to see this positive effect more pronounced in species with typical Angiosperm morphology, where the 3 apertures lend to the structural integrity of the pollen wall but minimize the locations more vulnerable to chemical penetration. Unfortunately, due to the extreme complexity of collecting sufficient amounts of insect pollinated species, free from contamination of other species or non-pollen plant material, inclusion of representatives of these taxa was not realistic.

Regardless of bead size used or the time interval needed to reach complete rupture, the resulting curve produced for time to complete rupture (Fig 1) follows a similar pattern in all species examined here. The species reach 33% relatively quickly in relation to the time needed reach higher levels of rupture. This means that the additional time required to reach complete rupture of all pollen grains also greatly increases the time previously ruptured pollen grains are exposed to collisions that will degrade the DNA. This produces a trade-off where, at some point in the bead milling process, the benefit of increased genetic material released from the pollen grain wall will be outweighed by the detriment of shearing of exposed DNA. This could partially explain why we see increased DNA quantity yet diminished sequencing results in the 100% rupture treatments, as evidenced by increased divergence from expected proportions. For this reason, as well as the logistical impossibility of reaching complete exine rupture with a mixed pollen sample, we recommend conservative effort be used to break the pollen wall prior to DNA extraction.

Additional support for being less aggressive in efforts to rupture pollen grain walls in a mixed sample is the comparatively successful results of our 0% rupture treatments with 1-hour lysis incubation to treatments with higher levels of rupture. Generally speaking, within each barcode or primer pairing, the same species were retrieved regardless of level of rupture. Higher rupture levels did increase the proportion of reads in species occurring in very low read numbers, but only increased species detection in a few cases. In addition to mechanical rupture, longer lysis incubation (2 hours) without exine rupture gave similar values of DNA quantity to the treatment with only one hour of lysis incubation, but in most cases numbers of paired reads were diminished to a point that necessitated elimination from analyses. The diminished results produced from a long lysis incubation are most evident in the 24-hour treatment, where DNA extractions produced quantities that were barely detectable and efforts at amplification failed. This indicates that in addition to taking precautions not to over-mill, caution should also be applied to not over expose samples to lysis buffer prior to DNA extraction and the lysis incubation step should not exceed one hour.

### Performance of barcodes/primer pairs

Our results indicate that all three common plant barcodes used here have the potential to be accurate in species retrieval in Illumina sequencing, although proportions of the species’ reads retrieved are likely to never be duplicated across different barcodes or accurately reflect the proportions of the starting materials.

The only consistent result we observed across all barcodes and primer pairs was the nearly complete exclusion of *Zea mays*. This could be due to the high GC content of this species, DNA regions with a GC content in excess of 60% require special PCR protocols to overcome strong secondary structures that interfere with primer annealing [50]. We augmented our PCR protocol with the addition of 5% DMSO, and were successful with amplification of *Z*. *mays* when no other species were contained in the sample, but this addition does not seem to be sufficient for returning this species from a mixed species sample. This behavior of *Z*. *mays* has also been shown in previous studies [21, 51, 52], where it was completely excluded or only recovered when the number of species in a mixture did not exceed two. Regardless of the reasons for exclusion of *Z*. *mays*, this result indicates the great importance of tests with mock communities in metabarcoding, particularly when the absence or presence of one or more particular species are critical to the experimental question.

The results for *rbcL* indicate the critical aspect of primer pair choice, especially when creation of internal primers are necessary to accommodate for the read length limitation of Illumina sequencing and when the potential species contained in the sample represent very diverse taxonomies. With one primer pairing (rbcLaF/rbclr506) we retrieved 12 of 14 species (exclusion of *Taxus baccata* and *Zea mays*) adding confidence to the reliability of *rbcL* in pollen metabarcoding studies. With the other primer pairing (rbcL2/rbclaR) however, only five of the 14 species contained within the mock community were retrieved. This result is in contrast with results of previous studies that examined mock communities [21, 51] where species retrieval was adequate, and further investigation is necessary to elucidate the reduced success we achieved here.

Our results for ITS1 also excluded several species, and only 9 of the 14 species were retrieved. This result is expected for the three Gymnosperms (*Abies concolor*, *Picea sp*., and *Taxus baccata*) in the sample, all of which have a read length too long for creation of paired reads, but could be added to analyses if only unidirectional reads are considered. Of more concern is the absence of *Tilia platyphyllos*, which cannot be explained by read length. This result indicates that ITS1 should be examined further with mock samples before it is widely utilized in pollen or plant metabarcoding, and it is not recommended for use in airborne samples that are likely to contain Gymnosperm species.

ITS2 resulted in the most consistent results with either primer pairing and returned the most species (all but *Z*. *mays*) indicating that this barcode gives reliable results across a high taxonomic breadth. These results demonstrate reliable species retrieval even with universal primers that contain instances of mismatches in several taxonomic groups. The plant specific ITS2 primers used here are recommended for use when a high taxonomic diversity is expected in a sample, as they lessen the potential for mismatches and may also result in an advantage for pollen samples with a high fungal contaminant component, such as airborne pollen samples.

### Evaluation of proportions

The resulting trends produced across the barcodes and/or primer pairings were inconsistent and for this reason we cannot untangle the treatment effect from several other forces that have the potential to change the proportion of reads returned.

The first possible source of deviation from equal proportions is the possibility of unequal starting material. Our counting method was based on well-established protocol [53] and several replications, however the possibility of errors in counting or small changes in quantities aliquoted while creating replications cannot be eliminated. This is likely the case with *Taxus baccata*, which was consistently under represented across all replicates, treatments, and barcodes. With the exception of *Zea mays*, whose absence can be explained by other factors, no other species displayed this trend (either consistently too low or too high), indicating that our methods for counting and distribution of near equal amounts of pollen grains was robust to error.

The intention of these experiments was to evaluate the effect of exine rupture on proportion of reads returned. Therefore, it was necessary to begin with a quantifiable ratio of broken to intact pollen grains rather than a predetermined amount of DNA from each species, based on individual quantities of DNA extraction and C-value of the species. We are aware that plant DNA barcodes are multi-copy markers with an unknown number of replicates in the pollen grains. Therefore, it is possible that the same quantity of starting material (ng/μl) of species in a mock community might not lead to equal sequence recovery. Furthermore, it is widely accepted that PCR bias strongly affects metabarcoding results in Eukarya.

Trends of individual species were not consistent across all barcodes and primer pairings and we are therefore unable to draw definitive conclusions of an ideal level of exine rupture, or how a species morphology and exine strength might affect its retrieval in Illumina sequencing. The deviation from expected proportions indicate however, with these barcodes and primer pairings, the 33% and 67% treatments produce the lowest deviation from expected proportions, with the exception of ITS1 which had the lowest deviation at 0%. These results strengthen the idea that exine rupture prior to DNA extraction does enhance results and aids in counteracting other sources of biases in downstream processes, however, this is not a linear relationship and extreme effort to lyse every pollen exine in a sample will also have detrimental effects on results.

Perhaps the greatest implication of our study is to further strengthen the idea that great care must be taken in attempting to extract conclusions about the quantity of starting material from the proportions of reads retrieved. The barcodes used here were adequate in retrieving all or a majority of species; however, results were completely inconsistent across treatments and barcodes in terms of proportions of these species retrieved. Our results indicate that Illumina sequencing can provide accurate information of presence or absence of a species, but should never be used to interpret quantities of those species within a sample.

## Conclusions

- Exine rupture does appear to enhance results, but over-processing can be detrimental for results. We recommend bead milling for 2–4 minutes in a 30 hz bead mill with 1–3 mm beads or a mix that contains these sizes, and a lysis incubation should not exceed 1 hour.
- When creating artificial pollen samples, we recommend a minimum of 100,000 pollen grains be used for starting material in order to obtain the quantities necessary for experimentation.
- The lack of trends for any species across barcodes illustrates the confounding problems associated with pollen metabarcoding using plant multi copy markers, as well as the role every component of the collection and laboratory processes can have on the proportion of reads returned from sequencing.
- The lack of clear trends recovered here further display the inability for pollen metabarcoding with Illumina sequencing to be indicative of the proportions of species present in the initial samples, however, species identification, except of the problematic *Zea mays*, is very successful.
